# The Prevalence and Impact of Evidence-Based Medications on Cardiovascular and Cerebrovascular Outcomes in Patients with Acute Coronary Syndrome Post-Revascularization in Oman

**DOI:** 10.3390/pharmacy11030079

**Published:** 2023-04-26

**Authors:** Dalia Ahmed Al-Hadithy, Juhaina Salim Al-Maqbali, Adil Al-Riyami, Mohammed Al Za’abi, Ibrahim Al-Zakwani

**Affiliations:** 1 Department of Pharmacology & Clinical Pharmacy, College of Medicine & Health Sciences, Sultan Qaboos University, Muscat 123, Oman; 2 Department of Pharmacy, Sultan Qaboos University Hospital, Muscat 123, Oman; 3 Department of Medicine, Sultan Qaboos University Hospital, Muscat 123, Oman; 4 Oman Medical Specialty Board, Muscat 123, Oman

**Keywords:** acute coronary syndrome, evidence-based medications, drug utilization, major adverse cardiovascular events, revascularization, Oman

## Abstract

Objectives: International cardiovascular guidelines recommend prescribing a combination of five evidence-based medications (EBM) for acute coronary syndrome (ACS) patients post-revascularization. This study aims to assess the prevalence and impact of prescribing the full (five medications) versus partial (four medications or fewer) EBM combination on major adverse cardiovascular and cerebrovascular events (MACCE) in patients with ACS post-revascularization. Methods: Data from patients with ACS who had revascularization between January 2016 and September 2021 were collected retrospectively. Patients were then followed up until March 2022 for MACCE. Results: The full EBM combination was prescribed to 70% of the patients. However, after taking into account the presence of contraindications and clinical factors, the actual adherence to the guidelines was 95%. Patients who received the full EBM combination were younger (58 versus 62 years; *p* = 0.0 and 3) and had lower rates of chronic kidney disease (11% versus 41%; *p* < 0.001) and heart failure (9% versus 20%; *p* = 0.012) when compared to patients who received the partial EBM. Compared to the partial EBM group, the full EBM group was associated with lower MACCE rates (54% versus 37%, *p* = 0.012). After employing the propensity score technique utilizing the 1:1 nearest neighbor matching method without replacement, the univariate findings were further re-affirmed with those on full EBMs (compared to those on partial EBMs) associated with a significant reduction in the MACCE rate (average treatment effect of −25%; 95% confidence interval: −10–−40%; *p* = 0.001). Conclusions: The full EBM utilization was significantly high in our setting and in line with international guidelines. The full EBM combination was predominantly prescribed in younger and less comorbid patients and was associated with lower MACCE rates. The findings were further reaffirmed by the propensity score matching method.

## 1. Introduction

Acute coronary syndrome (ACS) is a spectrum of myocardial ischemic states characterized by a sudden reduction in blood flow to the heart. They range from undifferentiated chest pain to unstable angina, and from non-ST-segment elevation myocardial infarction (NSTEMI) to ST-segment elevation myocardial infarction (STEMI) [1].

ACS is associated with significant morbidity and mortality due to the progression into an advanced disease state which may eventually lead to death [1,2]. For instance, ACS and sudden cardiac death contribute to the majority of deaths that are related to ischemic heart disease (IHD), which accounts for 1.8 million deaths per year worldwide [2]. In Oman, IHD was the leading cause of death in the period between 2009 and 2019 with a 14.2% increase and was associated with the highest age-standardized death and disability (DALY’s) rate per 100,000 in 2019 in comparison to other countries [3]. The most common risk factors that accounted for the increase in these DALYs in 2019 were high body mass index, followed by high blood pressure, high fasting blood glucose, and dietary risks. Older data from the Gulf registry of acute coronary events showed that hypertension was the most common risk factor among patients with ACS in Oman (53%), followed by diabetes mellitus (37%) and dyslipidemia (35%) [4]. Furthermore, patients who survive ACS remain at significant risk of future cardiovascular and ischemic events as well as mortality. Despite secondary prevention strategies, the rate of mortality, heart failure, and re-infarction remain high at 7.1%, 6.3%, and 4.4%, respectively, during the first two years post an ACS event [5].

The International guidelines (American College of Cardiology (ACC)/American Heart Association (AHA) and European Society of Cardiology (ESC)) have recommended prescribing a combination of five evidence-based cardiac medications (EBM) to patients with ACS who do not have contraindications to improve their clinical outcomes including morbidity and mortality [6,7,8,9]. The five medications of EBM include dual antiplatelet therapy (DAPT), an angiotensin-converting enzyme inhibitor (ACEI) or angiotensin II receptor blocker (ARB), a statin, and a beta-blocker [6,7,8,9]. Additionally, immediate or early revascularization with percutaneous coronary intervention (PCI) or coronary artery bypass grafts (CABG) is essential in certain patients following an ACS event [10,11]. 

Differences among the guidelines mainly lie in the strength of recommendations, the level of evidence, and the release date. For example, prescribing ACEI to STEMI patients who have heart failure, ejection fraction ≤ 40%, or those with anterior myocardial infarction (MI), and to NSTE-ACS (unstable angina and NSTEMI) patients with ejection fraction ≤ 40%, hypertension, diabetes mellitus, or chronic kidney disease is based on a Class I recommendation, and it is regarded as a Class II recommendation prescribed to all patients with ACS without contraindication according to the ACC/AHA and ESC guidelines; whereas the National Institute for Health and Care Excellence guidelines recommend prescribing ACEI indefinitely to patients with ACS once they are stabilized. However, adherence to these guidelines varies between countries and even among different hospitals within a country [12,13].

Furthermore, while each of the EBM has been shown to reduce major adverse cardiovascular and cerebrovascular events (MACCE) including all-cause mortality in published studies [12,13,14,15], only a few have examined the impact of the full EBM combination on MACCE in real-life settings in patients with ACS who have been revascularized. Therefore, this study aimed to evaluate the impact of the full EBM on MACCE including all-cause mortality and to determine the prevalence of prescribing this combination as well as the reasons behind their non-prescribing in patients with ACS who have undergone revascularization.

## 2. Methods

### 2.1. Study Design and Settings

This was a retrospective cohort study that was carried out by reviewing electronic records of patients admitted at Sultan Qaboos University Hospital (SQUH), Muscat, Oman, with a diagnosis of ACS and who had undergone revascularization. All data were obtained from the SQUH Information System (Trak Care).

### 2.2. Inclusion Criteria

All patients >18 years old who had undergone PCI or CABG and were diagnosed with ACS from 1 January 2016 to 30 September 2021 were included. Patients were then followed up for primary and secondary outcomes from their hospital discharge until the end of the study period (31 March 2022), or until their last reported follow-up.

### 2.3. Exclusion Criteria

Patients who did not follow-up at SQUH, those with missing data, and those who had MACCE within the first six months post an ACS event were excluded.

### 2.4. Data Collection

Baseline data included patients’ demographics, medical history, laboratory data, clinical presentation, and discharge medications. Patients’ clinical factors behind not prescribing EBM were also collected and included medical conditions, comorbidities, laboratory findings, or adverse drug reactions (ADRs). Follow-up data included laboratory investigations and subsequent data on MACCE that included re-infarction, heart failure, unplanned re-hospitalization for cardiac reasons, and all-cause mortality.

### 2.5. Evidence-Based Medication (EBM)

The EBM constituted of five classes of medications; aspirin, clopidogrel, beta-blocker (including; carvedilol, or bisoprolol), RAS blocker (including; lisinopril, irbesartan, or valsartan), and a statin (including; atorvastatin or rosuvastatin), or their equivalents and alternatives, if any. In patients with ACS with atrial fibrillation, oral anticoagulant with a single antiplatelet was considered equivalent to DAPT [16], and in NSTEMI patients with contraindications to beta-blockers, non-dihydropyridine calcium channel blockers (including verapamil or diltiazem) were considered as alternatives [6].

#### Full Versus Partial EBM

Patients were stratified into two groups based on the number of medications they received at discharge: full EBM group (five medications) and partial EBM group (four medications or fewer).

### 2.6. Study Outcomes

The primary outcome of this study was the proportion of patients who received the full EBM at discharge, while the secondary outcomes were the MACCE events which included re-infarction, heart failure, stroke, all-cause mortality, and re-admissions due to cardiac reasons. All-cause mortality was defined as death from any underlying cause that was reported in the hospital information system.

### 2.7. Sample Size

A previous study [17] reported EBM utilization of 69% in patients with ACS in the Arabian Gulf (Gulf COAST registry database that included four countries namely; Kuwait, Oman, United Arab Emirates, and Bahrain). We hypothesized that, based on a projected prevalence of around 80% for this academic tertiary center, a sample size of 243 patients with ACS with a margin error of 5% and a 95% confidence interval was needed. The sample size was further increased to 268 patients to account for any missing data.

### 2.8. Statistical Analysis

Descriptive statistics were used to describe the data. For categorical variables, frequencies and percentages were reported. Differences between groups were analyzed using Pearson’s χ^2^ tests (or Fisher’s exact tests for expected cells < 5). Normality for continuous variables was performed using the Kolmogorov–Smirnov test. For continuous variables, the mean and standard deviation were used to summarize the data while non-normally distributed variables were presented as the median and interquartile range and analyzed using the non-parametric test, Wilcoxon Mann–Whitney test. The association between the full (five medications) versus partial (four medications or fewer) EBM use with MACCE, adjusting for other confounding variables (age, heart failure, chronic kidney disease, hypertension, heart rate, diastolic blood pressure, estimated glomerular filtration rate, creatinine, and length of hospital stay) was evaluated using multivariate logistic regression. Additionally, the average treatment effect of full EBM use (compared to those on partial EBM regimen) was also performed using the propensity score technique utilizing the 1-1 nearest-neighbor matching method without replacement. A two-tailed level of significance was set at *p* < 0.05 level. Statistical analyses were carried out using STATA version 16.1 (StataCorp, 2013, Stata Statistical Software, College Station, TX, USA).

## 3. Results

The mean age of the cohort (n = 268) was 59 ± 12 years, 76% (n = 204) were male, 27% (n = 73) were active smokers or had a history of smoking, and 5.2% (n = 14) were alcohol consumers. Patients with MI accounted for 90% (n = 241) of the cohort, with 42% (n = 112) of the patients diagnosed with STEMI, 48% (n = 129) had NSTEMI, and 10% (n = 27) had unstable angina. The most frequent comorbidity was hypertension 68% (n = 181), followed by diabetes mellitus 59% (n = 157) and dyslipidemia 55% (n = 148) (Table 1).

As shown in Table 1, 70% (n = 188) of the patients were prescribed the full EBM while 30% (n = 80) were on the partial EBM. Patients in the full EBM group, compared to the partial EBM cohort, were significantly younger (58 versus 62 years; *p* = 0.03), had significantly fewer comorbidities, namely chronic kidney disease (11% versus 41%; *p* < 0.001), and heart failure (9% versus 20%; *p* = 0.012), but had a significantly higher prevalence of hypertension (72% versus 58%; *p* = 0.022). However, the partial EBM group had a significantly longer length of hospital stay compared to the full EBM cohort (3 versus 2 days; *p* < 0.001).

**Figure 1:**
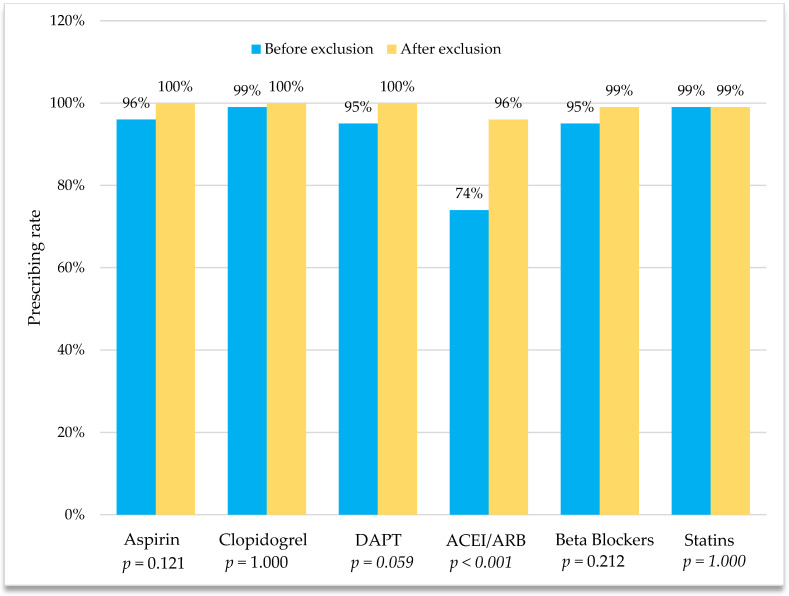
Prescribing rates of evidence-based medications (EBMs) before and after excluding patients with contraindications and clinical reasons for not prescribing them. DAPT, dual antiplatelet therapy; ACEI, angiotensin-converting enzyme inhibitor; ARB, angiotensin receptor blocker; n = 268 (before excluding those with clinical reasons); n = 187 (after excluding those with clinical reasons).

Figure 1 demonstrates the rate of EBM utilization and DAPT prescribing patterns. DAPT was prescribed in 95% (n = 254) of patients, which consisted of aspirin and clopidogrel prescribed together. Clopidogrel and statins were prescribed the most, with a rate of 99% (n = 266) for each medication independently, followed by aspirin (96%; n = 256), beta-blockers (96%; n = 257), and RAS blockers (74%; n = 199). Of note, about 7.1% (n = 19) of patients received oral anticoagulants. Out of those that received oral anticoagulants, 32% (6/19) also received DAPT, and the remaining 68% (13/19) were prescribed anticoagulants with a single antiplatelet as a replacement for DAPT. In total, more than 99% (n = 267) of the patients received either DAPT or a single antiplatelet with an oral anticoagulant, and only one patient received clopidogrel (Table 2).

Table 3 outlines the reasons behind the non-prescribing of EBM. In the partial EBM group, the most frequently not prescribed medication class was RAS blockers (26%), followed by beta-blockers (4%), and statins (1%). The presence of contraindications or clinical reasons accounted for 88% (72/82) of the explanations behind the non-prescribing of the full EBM. The most common reason for not prescribing beta-blockers was bradycardia (60%), while RAS blockers were mostly not prescribed due to hypotension (42%) and impaired kidney function (38%). Among patients who were not on RAS blockers due to impaired kidney function, 42% (n = 29) received an isosorbide dinitrate and hydralazine combination. The patients who were not on statins due to muscle pain received the proprotein convertase subtilisin/kexin type 9 (PCSK9) inhibitor, alirocumab, at discharge. Patients who were in the full EBM group but did not receive aspirin (n = 9), clopidogrel (n = 1), or a beta-blocker (n = 1) were discharged with an equivalent or an alternative instead. After excluding those with clinical reasons for not prescribing EBMs, the prescribing prevalence was re-estimated to be 95% (177/187) (Figure 1).

During the median follow-up period of 2 (1.1–2.9) years, the rates of re-admissions due to cardiac reasons, recurrent MI, heart failure, stroke, and all-cause mortality were 40% (n = 106), 15% (n = 39), 13% (n = 34), 3% (n = 7), and 3% (n = 7), respectively, resulting in an overall MACCE rate of 42% (n = 113). The full EBM group had a statistically significantly lower risk of MACCE compared to the partial EBM group (37% versus 54%, *p* = 0.012). Table 4 outlines the covariate balance between the EBM groups before and after the propensity score matching method. There were largely no significant differences among the groups with regards to demographic and clinical characteristics as most of the standardized mean differences were below the threshold cut-off of 25 or below as recommended by Ruben DB [18]. Propensity score matching largely balanced the groups in all the covariates except for chronic kidney disease, serum creatinine, and length of hospital stay.

**Figure 2:**
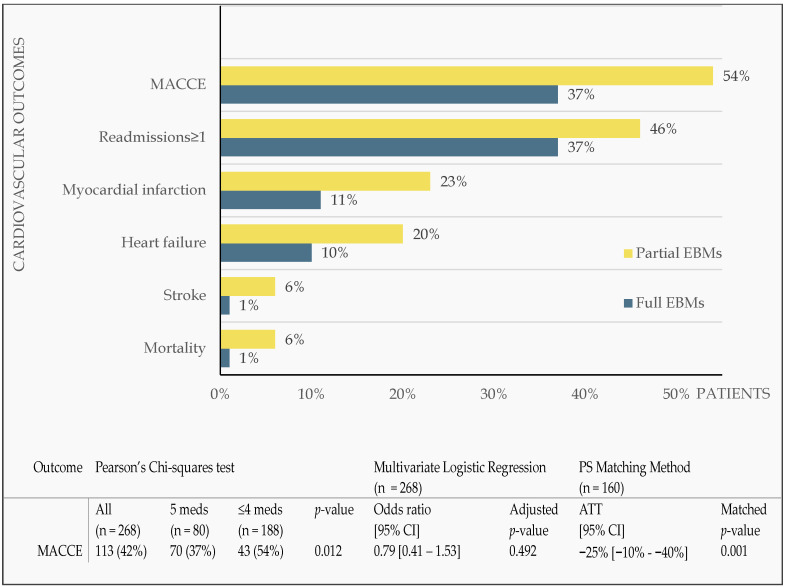
Impact of full and partial evidence-based medications on major adverse cardiovascular events using univariate analysis. Meds, medications; PS, propensity score; CI, confidence interval; ATT, average treatment effect on the treated; MACCE, major adverse cardiovascular and cerebrovascular events; n = 268.

Figure 2 shows the various types of MACCE. After adjusting for patients’ clinical factors, the multivariate logistic regression models demonstrated no statistically significant differences in the risk of MACCE between the two groups (adjusted odds ratio (aOR) 0.79; 95% confidence interval (CI): 0.41–1.53; *p* = 0.492). However, the propensity score matching method re-affirmed the univariate findings in that those on EBMs (five medications compared to those on ≤four medications) were associated with a significant reduction in the MACCE rate of 25% (95% CI: 10–40%; *p* = 0.001).

## 4. Discussion

Two main findings were demonstrated in this study. Firstly, 70% of our cohort received the five EBMs at discharge (this increased to 95% after accounting for contraindications of EBM use), and the prescribing of these medications was mostly observed in younger patients and those with fewer comorbidities. Secondly, the univariant analysis showed a significant reduction in MACCE in the full EBM group compared to the partial EBM group which was further reaffirmed by the propensity score matching method.

The initial apparent prescribing rate in our study was broadly comparable with other studies that frequently reported around 70% [17,19,20], and higher than a few others (36.2% and 46.2%) [21,22]. However, on removing contraindications or clinical reasons for not prescribing EBM, a 95% prescribing rate was obtained. A substantial number of studies have evaluated the prescribing patterns of EBM use in patients with ACS in their respective hospitals with a significant variation in prescribing rates [17,20,21,22,23,24,25]. According to the Gulf COAST registry database, EBM utilization reached 69% in patients with ACS in the Arabian Gulf region [17]. This finding was estimated without considering the presence of contraindications that might have hindered the prescribing of some medications. However, the reasons for not prescribing EBM use were explored in our study.

In line with our findings, among all EBM, RAS blockers were the least frequent medications to be prescribed across multiple studies, including those in the Middle East (Gulf: 67% for ACEIs and 15% for ARBs; Iraq: 69.5% for RAS blockers) [17,25]. A possible explanation is that the presence of marginal clinical parameters and perceived contraindications to the use of this medication class is common, particularly in patients with ACS. For instance, hypotension and impaired kidney function accounted for the majority of gaps in RAS blockers prescribed in the current study. It is worth noting that nearly half of the patients who were not prescribed RAS blockers received a combination of hydralazine and isosorbide dinitrate. However, this practice seems to be an extrapolation of the heart failure guidelines [26]. This opens a gap in the literature and lays the ground for future work to test the hypothesis of whether the combination of isosorbide dinitrate and hydralazine can be considered as an alternative to RAS blockers in patients with ACS. Another reason could be the fact that there is a difference in the strength of recommendation and whether RAS blockers should be prescribed to all patients with ACS or only to those at high risk [6,7].

In our study, the vast majority of patients (95%) received DAPT and almost all the remaining patients received an antiplatelet with an anticoagulant instead. These results reflect regular updating and adherence to ACS prescribing guidelines. Given that platelet activation is an important step in the pathogenesis of post-ACS and post-PCI ischemic events, the use of DAPT in the management of patients with ACS, specifically those undergoing revascularization, is crucial and has been widely shown to reduce MACCE in ACS and PCI patients [10,11,15,27].

Our analysis showed that the prescribing rate of ACE-I/ARBs increased from 74% to 96% after considering the presence of clinical factors/contraindications. With regards to the other medications, the small differences in the rates prove that these medications are already being prescribed to the majority of the patients regardless of the presence of contraindications. The number of medications prescribed was significantly impacted by the patient’s age and medical history. For instance, older patients and those with chronic kidney disease or heart failure received fewer EBMs upon discharge. Although this group of patients carries a greater risk of having subsequent cardiovascular events and, therefore, could benefit the most from secondary prevention therapy [6], these findings are unsurprising and the trend of lower EBM prescribing in high-risk patients has been reported in prior published research [22]. We believe that the reason behind the low prescribing of these medications is the presence of clinical factors/contraindications that hinder the use of full EBM. In line with previous findings from Oman [4], patients with ACS were found to be younger in this study compared to other countries [21,22,28].

In addition to completing cardiovascular education programs and integrated care implementation, one of the strategies to reduce the gap between guidelines’ recommendations and actual prescribing rates could be the use of a pre-discharge medications checklist, which has been shown to significantly increase the prescribing rates of each EBM component indicated for MI patients [29].

Using univariant analysis, the full EBM group was associated with a lower MACCE rate which is almost in line with most studies, and revealed that EBM use has a positive impact on mortality [17,19,22,24], and in a few studies, on morbidity as well [30]. However, we observed a non-significant difference in MACCE reduction between both EBM groups after multivariate logistic regression. Possible explanations for this include, first and foremost, sample size and power of the study which were calculated based on EBM prevalence rather than the more complicated multivariate regression on MACCE which would require a larger sample size. Second, almost all the patients in the comparison group received four medications, and only two patients received three medications. The partial EBM has also been associated with lower MACCE in prior studies [17] and this could have explained the non-significant findings. Furthermore, some studies which evaluated different EBM combinations showed that combinations with and without ACEI resulted in similar outcomes in ACS [12,19]. Finally, the use of undocumented co-medications might have also played a role. For example, in patients with diabetes mellitus, which constituted more than half of this study’s cohort, novel antihyperglycemic agents such as sodium-glucose cotransporter-2 (SGLT2) inhibitors and glucagon-like peptide-1 (GLP-1) agonists have been shown to significantly reduce different MACCE in patients with established cardiovascular disease [31]. Of note, the univariate findings were further reaffirmed by the propensity score matching method.

To our knowledge, this is the first study in Oman to identify the reasons for not prescribing the full EBM and the impact of EBM on MACCE in patients with ACS undergoing revascularization. This study is not without limitations. First, the sample size calculations were based on the prevalence of EBM use rather than MACCE. Furthermore, there is a possibility of the presence of unmeasured confounding variables that might have impacted the findings due to the retrospective nature of the study design. Second, the generalizability of the data to the whole population cannot be surmised with certainty even though the study has been conducted in a major academic center in Oman. Finally, it is not beyond the scope of this study to document optimal doses or patients’ adherence which could also have confounded our findings.

## 5. Conclusions

Based on the study findings, it can be concluded that EBM utilization, especially clopidogrel and statins, was substantially high, with the vast majority of patients with ACS receiving the full EBM at discharge after considering the reasons for not prescribing full EBM combinations. This should encourage the continuation of the current practice at SQUH. However, there is a need to improve the documentation and descriptions of patients’ clinical characteristics and medication use. The significant advantages of using the five EBMs in univariate analysis were further reaffirmed by the propensity score matching method. Given the study’s inherent design limitations, the findings should be deduced with caution. Further research is needed to corroborate the findings.

## Figures and Tables

**Table 1:** Demographic and baseline clinical and laboratory characteristics stratified by evidence-based medication combinations in acute coronary syndrome patients.

Characteristic, n (%) Unless Specified Otherwise	All (n = 268)	EBM		*p*-Value
		Partial (n = 80)	Full (n = 188)	
Demographic				
Age, mean ± SD, years	59 ± 12	62 ± 14	58 ± 12	0.03
Female gender	64 (24%)	20 (25%)	44 (23%)	0.779
Smoker	73 (27%)	19 (24%)	54 (29%)	0.403
Medical history				
Atrial fibrillation	16 (6%)	7 (9%)	9 (5%)	0.21
Heart failure	33 (12%)	16 (20%)	17 (9%)	0.01
Stroke	13 (5%)	6 (8%)	7 (4%)	0.18
Transient ischemic attack	7 (3%)	4 (5%)	3 (2%)	0.11
Chronic kidney disease	53 (20%)	33 (41%)	20 (11%)	<0.001
Diabetes mellitus	157 (59%)	47 (59%)	110 (59%)	0.97
Hypertension	181 (68%)	46 (58%)	135 (72%)	0.02
Dyslipidemia	148 (55%)	42 (53%)	106 (56%)	0.55
Clinical parameters at presentation				
Heart rate, mean ± SD, beats/min	72 ± 12	69 ± 12	74 ± 12	<0.001
Systolic BP, mean ± SD, mmHg	125 ± 19	125 ± 23	125 ± 18	0.81
Diastolic BP, mean ± SD, mmHg	69 ± 14	65 ± 13	71 ± 14	<0.001
LVEF, mean ± SD, %	52 ± 11	53 ± 12	51 ± 11	0.23
Laboratory parameters at the presentation				
HbA1c, mean ± SD, %	7.0 ± 2.0	8.0 ± 2.0	7.0 ± 2.0	0.13
Potassium, mean ± SD, mmol/L	4.0 ± 0.4	4.0 ± 0.5	4.0 ± 0.4	0.91
Creatinine, mean ± SD, μmol/L	101 ± 7	144 ± 12	82 ± 25	<0.001
eGFR, mean ± SD, mL/min/1.73 m^2^	72 ± 22	60 ± 28	77 ± 15	<0.001
Discharge diagnosis				
NSTEMI	129 (48%)	44 (55%)	85 (45%)	0.14
STEMI	112 (42%)	27 (34%)	85 (45%)	0.08
Unstable angina	27 (10%)	8 (10%)	19 (10%)	0.88
LOS during admission, median (IQR) days	2 (2–4)	3 (2–5)	2 (2–3)	<0.001

EBM, evidence-based medication (five medication combinations of aspirin, clopidogrel, angiotensin-converting enzyme inhibitor or angiotensin receptor blocker, beta-blocker, and statin, or their alternatives are considered full EBM); SD, standard deviation; BP, blood pressure; LVEF, left ventricular ejection fraction; HbA1c, glycated hemoglobin; eGFR, estimated glomerular filtration rate; NSTEMI, non-ST elevation myocardial infarction; STEMI, ST elevation myocardial infarction; LOS, length of stay; IQR, interquartile range.

**Table 2:** Medication utilization stratified by evidence-based medication combinations in acute coronary syndrome patients.

Characteristic, (%)	All (n = 268)	Full EBM		*p*-Value
		No (n = 80)	Yes (n = 188)	
*Discharged medications*				
DAPT	254 (95%)	79 (95%)	178 (95%)	1
Aspirin	256 (96%)	77 (96%)	179 (95%)	1
Clopidogrel	266 (99%)	79 (99%)	187 (99%)	0.509
Oral anticoagulant	19 (7%)	5 (6%)	14 (7%)	1
Warfarin	11 (4%)	4 (5%)	7 (4%)	0.738
DOAC	8 (3%)	1 (1%)	7 (4%)	0.442
ACEI/ARB	199 (74%)	11 (14%)	188 (100%)	<0.001
ACEI (Lisinopril)	162 (60%)	9 (11%)	153 (81%)	<0.001
ARB	37 (14%)	2 (3%)	35 (19%)	<0.001
Irbesartan	25 (9%)	1 (1%)	24 (13%)	0.002
Valsartan	12 (5%)	1 (1%)	11 (6%)	0.116
Beta-blocker ^a^	257 (95%)	70 (88%)	187 (99%)	<0.001
Bisoprolol	217 (81%)	60 (75%)	157 (84%)	0.104
Carvedilol	39 (15%)	10 (13%)	29 (15%)	0.534
Statin	266 (99%)	78 (98%)	188 (100%)	0.088
Atorvastatin	242 (90%)	73 (91%)	169 (90%)	0.731
Rosuvastatin	24 (9%)	5 (6%)	19 (10%)	0.36
Hydr + isdn	48 (18%)	31 (39%)	17 (9%)	<0.001
CCB (Amlodipine)	61 (23%)	18 (23%)	43 (23%)	0.947
Spironolactone	19 (7%)	6 (8%)	13 (7%)	0.864

EBM, evidence-based medication (five medication combinations of aspirin, clopidogrel, angiotensin-converting enzyme inhibitor or angiotensin receptor blocker, beta-blocker, and statin, or their alternatives are considered full EBM); DAPT, dual anti-platelet therapy (aspirin and clopidogrel); DOAC, direct oral anti-coagulants; ACEI, angiotensin-converting enzyme inhibitor; ARB, angiotensin receptor blockers; Hydr, hydralazine; isdn, isosorbide dinitrate; CCB, calcium channel blocker; ^a^ one patient was on atenolol in the evidence-based medications group.

**Table 3:** Reasons for the non-prescribing of each medication class in patients not receiving full evidence-based medications.

Medication, n (%)	Not on Medication n = 82	Reasons behind Non-Prescribing	n (%)
Clopidogrel	1 (<1%)	1. Bleeding event	1 (100%)
Beta-blockers	10 (4%)	1. Bradycardia	6 (60%)
		2. No documented reason	2 (20%)
		3. Hypotension	1 (10%)
		4. Asthma	1 (10%)
RAS blockers	69 (26%)	1. Hypotension	29 (42%)
		2. Impaired kidney function	26 (38%)
		3. Hyperkalemia	6 (9%)
		4. No documented reason	7 (10%)
		5. Cough	1 (1%)
Statins	2 (1%)	1. Muscle pain	1 (50%)
		2. No documented reason	1 (50%)

RAS, renin-angiotensin system; two patients were not taking two evidence-based medications simultaneously.

**Table 4:** Covariate balance across the evidence-based medication combination groups before and after the propensity score (PS) technique using the one-to-one nearest-neighbor matching method without replacement.

Characteristic	Original Cohort			PS Matched Cohort		
	5 Meds	≤4 Meds	SMD	5 Meds	≤4 Meds	SMD
	(n = 188)	(n = 80)		(n = 80)	(n = 80)	
Age, mean, years	58.41	61.84	−0.27 *	59.96	61.84	−0.15
Heart failure, %	9%	20%	−0.31 *	11%	20%	−0.25
Chronic kidney disease, %	11%	41%	−0.74 *	20%	41%	−0.51 *
Hypertension, %	72%	58%	0.31 *	49%	58%	−0.18
Heart rate, mean, bpm	73.88	69.18	0.39 *	71.97	69.18	0.23
Diastolic BP, mean, mmHg	70.74	64.91	0.43 *	67.06	64.91	0.16 *
eGFR, mean, ml/min/1.73 m^2^	76.80	59.59	0.76 *	72.61	59.59	0.57 *
Creatinine, mean, μmol/L	82.07	143.66	−0.68 *	88.89	43.66	−0.60 *
Length of stay, mean, days	2.91	5.40	−0.45 *	3.18	5.40	−0.40 *

Meds, medications; SMD, standardized mean difference; bpm, beats per minute; BP, blood pressure; eGFR, estimated glomerular filtration rate. Evidence-based medications (include aspirin, clopidogrel, angiotensin-converting enzyme inhibitor or angiotensin receptor blocker, beta-blocker, and statin).* SMD values above 0.25 are indicative of covariate imbalance as reported by Ruben DB [18].

## Data Availability

The authors have complete access to the study data. The data are not accessible to readers.
